# Crystal structure of (*E*)-1-(4′-meth­oxy-[1,1′-biphen­yl]-4-yl)-3-(3-nitro­phen­yl)prop-2-en-1-one

**DOI:** 10.1107/S2056989014025110

**Published:** 2015-01-01

**Authors:** T. Vidhyasagar, K. Rajeswari, D. Shanthi, M. Kayalvizhi, G. Vasuki, A. Thiruvalluvar

**Affiliations:** a Department of Chemistry, Annamalai University, Annamalai Nagar 608 002, Tamilnadu, India; b Department of Physics, Kunthavai Naachiar Government Arts College (W) (Autonomous), Thanjavur 613 007, Tamilnadu, India; c Postgraduate Research Department of Physics, Rajah Serfoji Government College (Autonomous), Thanjavur 613 005, Tamilnadu, India

**Keywords:** crystal structure, chalcones, C—H⋯O hydrogen bonding, main-residue disorder

## Abstract

The title compound, C_22_H_17_NO_4_, crystallizes with two independent mol­ecules (*A* and *B*) in the asymmetric unit. Each mol­ecule exists as an *E* isomer with C—C=C—C torsion angles of −175.69 (17) and −178.41 (17)° in *A* and *B*, respectively. In mol­ecule *A*, the planes of the terminal benzene rings are twisted by an angle of 26.67 (10)°, while the biphenyl unit is non-planar, the dihedral angle between the rings being 30.81 (10)°. The dihedral angle between the nitro­phenyl ring and the inner phenyl ring is 6.50 (9)°. The corresponding values in mol­ecule *B* are 60.61 (9), 31.07 (8) and 31.05 (9)°. In the crystal, mol­ecules are arranged in a head-to-head manner, with the 3-nitro­phenyl groups nearly parallel to one another. The *A* and *B* mol­ecules are linked to one another *via* C—H⋯O hydrogen bonds, forming chains lying parallel to (-320) and enclosing *R*_2_^2^(10) and *R*_2_^2^(12) ring motifs. The meth­oxy group in both mol­ecules is positionally disordered with a refined occupancy ratio of 0.979 (4):0.021 (4) for mol­ecule *A* and 0.55 (4):0.45 (4) for mol­ecule *B*.

## Abstract 2

The title compound crystallized with two independent mol­ecules in the asymmetric unit. In the crystal, they are linked to one another, forming chains enclosing 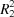
(10) and 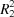
(12) ring motifs.

## Chemical context   

Chalcones have been reported to possess many inter­esting pharmacological activities (Dhar, 1981 ▸), including anti-inflammatory, anti­microbial, anti­fungal, anti­oxidant, cytotoxic, anti­tumor and anti­cancer activities (Dimmock *et al.*, 1999 ▸; Satyanarayana *et al.*, 2004 ▸). The effect of new biphenyl chalcone derivatives against gamma-radiation-induced oxidative stress markers in *E. coli* K 12, and the evaluation of their anti­microbial activities have been reported (Darshan Raj *et al.*, 2013 ▸).
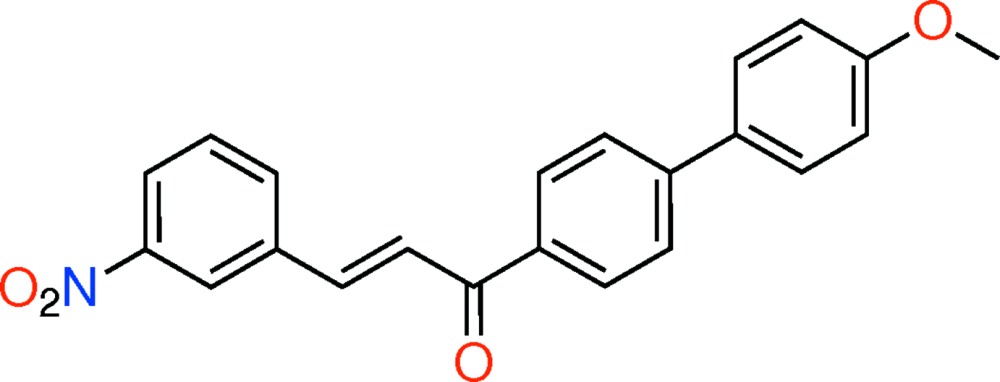


## Structural commentary   

**Figure 1:**
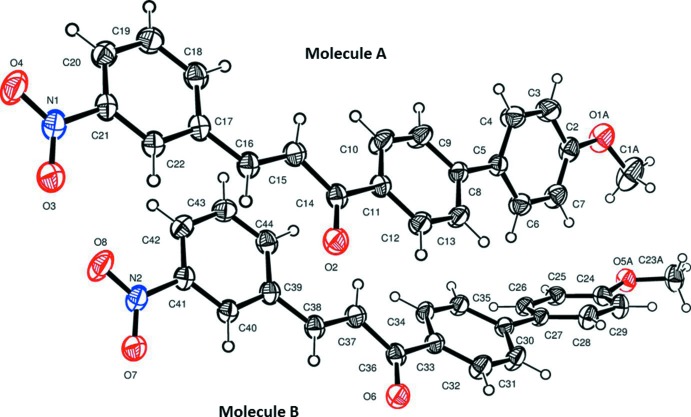
The mol­ecular structure of the two independent mol­ecules (*A* and *B*) of the title compound, with atom labelling. Displacement ellipsoids are drawn at the 30% probability level (the minor components of the disordered meth­oxy groups are not shown).

The title compound, Fig. 1 ▸, crystallizes with two independent mol­ecules (*A* and *B*) in the asymmetric unit. Each mol­ecule exists as an E isomer with the C17—C16—C15—C14 and C39—C38—C37—C36 torsion angles being −175.69 (17) and −178.41 (17)°, respectively. In mol­ecule *A*, the terminal benzene rings (C2–C7) and (C17–C22) are twisted by an angle of 26.67 (10)°, while the biphenyl rings (C2–C7 and C8–C13) are non-planar, the dihedral angle being 30.81 (10)°. The dihedral angle between rings (C8–C13) and (C17–C22) is 6.50 (9)°. The corresponding dihedral angles in mol­ecule *B* are (C24–C29 and C39–C44) 60.61 (9), (C30—C35 and C24–C29) 31.07 (8) and (C30–C35 and C39–C44) 31.05 (9)°.

## Supra­molecular features   

**Figure 2:**
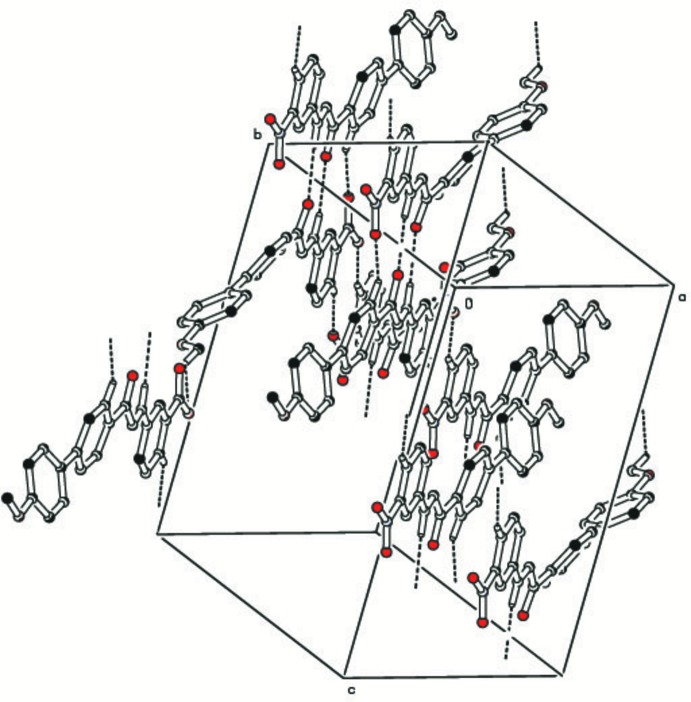
A partial view along the *a* axis of the crystal packing of the title compound, showing the C—H⋯O hydrogen bonds (dashed lines; see Table 1 ▸ for details; H atoms not involved in hydrogen bonding have been omitted for clarity).

In the crystal, mol­ecules *A* and *B* lie head-to head almost parallel to one another. They are linked *via* C—H⋯O hydrogen bonds, forming chains lying parallel to (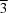
20) and enclosing 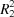
(10) and 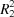
(12) ring motifs (Table 1 ▸ and Fig. 2 ▸).

## Database survey   

A search of the Cambridge Structural Database (Version 5.35, May 2014; Groom & Allen, 2014 ▸) for the substructure 1-([1,1′-biphen­yl]-4-yl)-3-phenyl­prop-2-en-1-one revealed the presence of a number of similar compound, including (2*E*)-3-(biphenyl-4-yl)-1-(4,4′′-di­fluoro-5′-meth­oxy-1,1′:3′,1′′-ter­phen­yl-4′-yl)prop-2-en-1-one (Betz *et al.*, 2013 ▸), (*E*)-1-([1,1′-biphen­yl]-4-yl)-3-(2-methyl­phen­yl)prop-2-en-1-one (Shanthi *et al.*, 2014 ▸) and a structure very similar to the title compound, *viz*. 1-(4′-methyl­biphenyl-4-yl)-3-(3-nitro­phen­yl)prop-2-en-1-one (Varghesse *et al.*, 2014 ▸). In this last compound, the biphenyl rings are inclined to one another by 38.02 (15)°, while the inner phenyl ring is inclined to the nitro­phenyl ring by 5.29 (16)°. These values are similar to those observed for mol­ecule *A* of the title compound, *viz*. 30.8 (1) and 6.50 (9)°, respectively.

## Synthesis and crystallization   

A mixture of 4-acetyl-4′-meth­oxy­biphenyl (3.59 g, 10 mmol) and 3-nitro benzaldehyde (1.07 g, 10 mmol) in ethanol (25 ml) in the presence of NaOH (10 ml 30%) was heated in a water bath for 30 min. and then allowed to cool. The solid that separated was filtered and recrystallized from ethanol. The yellow-coloured crystals of the title compound used for the X-ray diffraction study were grown by slow evaporation from acetone (yield: 1.48 g; 70%).

## Refinement   

Crystal data, data collection and structure refinement details are summarized in Table 2 ▸. All H-atoms were positioned geometrically and allowed to ride on their parent atoms, with C—H = 0.93 − 0.96 Å with *U*_iso_(H) = 1.5*U*_eq_(C) for methyl H atoms and = 1.2*U*_eq_(C) for other H atoms. The refined occupancy ratios for the disordered meth­oxy groups are 0.979 (4):0.021 (4) for atoms O1*A*/O1*B* and C1*A*/C1*B* in mol­ecule *A* and 0.55 (4):0.45 (4) for atoms O5*A*/O5*B* and C23*A*/C23*B* in mol­ecule *B*.

## Supplementary Material

Crystal structure: contains datablock(s) I. DOI: 10.1107/S2056989014025110/su5021sup1.cif

Structure factors: contains datablock(s) I. DOI: 10.1107/S2056989014025110/su5021Isup2.hkl

Supporting information file. DOI: 10.1107/S2056989014025110/su5021Isup3.cdx

Supporting information file. DOI: 10.1107/S2056989014025110/su5021Isup4.cml

CCDC reference: 1034620

Additional supporting information:  crystallographic information; 3D view; checkCIF report

## Figures and Tables

**Table 1:** Hydrogen-bond geometry (, )

*D*H*A*	*D*H	H*A*	*D* *A*	*D*H*A*
C12H12O7^i^	0.93	2.42	3.185(2)	139
C16H16O6^i^	0.93	2.48	3.349(2)	156
C20H20O4^ii^	0.93	2.52	3.370(2)	151
C23*A*H23*A*O4^iii^	0.96	2.55	3.356(14)	142
C38H38O2^i^	0.93	2.49	3.361(2)	156
C42H42O8^iv^	0.93	2.42	3.291(2)	156

Symmetry codes: (i) 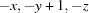; (ii) 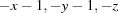; (iii) 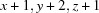; (iv) 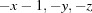.

**Table 2:** Experimental details

Crystal data	
Chemical formula	C_22_H_17_NO_4_
*M*_r_	359.37
Crystal system, space group	Triclinic, *P* 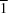
Temperature (K)	293
*a*, *b*, *c* ()	10.1924(3), 10.8732(3), 16.9675(6)
, , ()	97.926(2), 93.711(2), 107.729(2)
*V* (^3^)	1762.61(10)
*Z*	4
Radiation type	Mo *K*
(mm^1^)	0.09
Crystal size (mm)	0.35 0.35 0.30

Data collection	
Diffractometer	Bruker Kappa APEXII CCD
Absorption correction	Multi-scan (*SADABS*; Bruker, 2004 ▸)
*T*_min_, *T*_max_	0.833, 1.000
No. of measured, independent and observed [*I* > 2(*I*)] reflections	39509, 10197, 4932
*R*_int_	0.034
(sin /)_max_ (^1^)	0.704

Refinement	
*R*[*F* ^2^ > 2(*F* ^2^)], *wR*(*F* ^2^), *S*	0.062, 0.213, 1.05
No. of reflections	10197
No. of parameters	525
No. of restraints	122
H-atom treatment	H-atom parameters constrained
_max_, _min_ (e ^3^)	0.38, 0.18

Computer programs: *APEX2*, *SAINT* and *XPREP* (Bruker, 2004 ▸), *SHELXS2013* and *SHELXL2014* (Sheldrick, 2008 ▸), *ORTEP-3 for Windows* (Farrugia, 2012 ▸) and *PLATON* (Spek, 2009 ▸).

## supplementary crystallographic information

### Crystal data

**Table:**

C_22_H_17_NO_4_	*Z* = 4
*M**_r_* = 359.37	*F*(000) = 752
Triclinic, *P*1	*D*_x_ = 1.354 Mg m^−^^3^
Hall symbol: -P 1	Melting point: 446.6 K
*a* = 10.1924 (3) Å	Mo *K*α radiation, λ = 0.71073 Å
*b* = 10.8732 (3) Å	Cell parameters from 8416 reflections
*c* = 16.9675 (6) Å	θ = 2.1–29.6°
α = 97.926 (2)°	µ = 0.09 mm^−^^1^
β = 93.711 (2)°	*T* = 293 K
γ = 107.729 (2)°	Block, yellow
*V* = 1762.61 (10) Å^3^	0.35 × 0.35 × 0.30 mm

### Data collection

**Table:**

Bruker Kappa APEXII CCD diffractometer	10197 independent reflections
Radiation source: fine-focus sealed tube	4932 reflections with *I* > 2σ(*I*)
Graphite monochromator	*R*_int_ = 0.034
ω and φ scan	θ_max_ = 30.0°, θ_min_ = 2.1°
Absorption correction: multi-scan (*SADABS*; Bruker, 2004)	*h* = −14→14
*T*_min_ = 0.833, *T*_max_ = 1.000	*k* = −15→15
39509 measured reflections	*l* = −23→23

### Refinement

**Table:**

Refinement on *F*^2^	Primary atom site location: structure-invariant direct methods
Least-squares matrix: full	Secondary atom site location: difference Fourier map
*R*[*F*^2^ > 2σ(*F*^2^)] = 0.062	Hydrogen site location: inferred from neighbouring sites
*wR*(*F*^2^) = 0.213	H-atom parameters constrained
*S* = 1.05	W = 1/[Σ^2^(*F*O^2^) + (0.1041*P*)^2^ + 0.077*P*] where *P* = (*F*O^2^ + 2*F*C^2^)/3
10197 reflections	(Δ/σ)_max_ < 0.001
525 parameters	Δρ_max_ = 0.38 e Å^−^^3^
122 restraints	Δρ_min_ = −0.18 e Å^−^^3^

### Special details

**Table:**

Geometry. All e.s.d.'s (except the e.s.d. in the dihedral angle between two l.s. planes) are estimated using the full covariance matrix. The cell e.s.d.'s are taken into account individually in the estimation of e.s.d.'s in distances, angles and torsion angles; correlations between e.s.d.'s in cell parameters are only used when they are defined by crystal symmetry. An approximate (isotropic) treatment of cell e.s.d.'s is used for estimating e.s.d.'s involving l.s. planes.
Refinement. Refinement on *F*^2^ for ALL reflections except those flagged by the user for potential systematic errors. Weighted *R*-factors *wR* and all goodnesses of fit *S* are based on *F*^2^, conventional *R*-factors *R* are based on *F*, with *F* set to zero for negative *F*^2^. The observed criterion of *F*^2^ > σ(*F*^2^) is used only for calculating -*R*-factor-obs *etc*. and is not relevant to the choice of reflections for refinement. *R*-factors based on *F*^2^ are statistically about twice as large as those based on *F*, and *R*-factors based on ALL data will be even larger.

### Fractional atomic coordinates and isotropic or equivalent isotropic displacement parameters (Å^2^)

**Table:**

	*x*	*y*	*z*	*U*_iso_*/*U*_eq_	Occ. (<1)
C2	0.3176 (2)	0.86074 (18)	0.59986 (11)	0.0600 (5)	
C3	0.2833 (2)	0.72837 (19)	0.59925 (11)	0.0719 (6)	
H3	0.2900	0.6968	0.6471	0.086*	
C4	0.2389 (2)	0.64194 (18)	0.52814 (11)	0.0655 (5)	
H4	0.2173	0.5526	0.5288	0.079*	
C5	0.22572 (17)	0.68477 (15)	0.45580 (10)	0.0450 (4)	
C6	0.25966 (19)	0.81819 (16)	0.45774 (11)	0.0566 (5)	
H6	0.2521	0.8502	0.4102	0.068*	
C7	0.3050 (2)	0.90599 (17)	0.52935 (11)	0.0648 (5)	
H7	0.3268	0.9955	0.5292	0.078*	
C8	0.17944 (17)	0.58949 (15)	0.38024 (9)	0.0443 (4)	
C9	0.0900 (2)	0.46430 (17)	0.37875 (11)	0.0654 (6)	
H9	0.0564	0.4406	0.4260	0.078*	
C10	0.0495 (2)	0.37390 (17)	0.30937 (11)	0.0649 (6)	
H10	−0.0106	0.2907	0.3107	0.078*	
C11	0.09643 (17)	0.40462 (15)	0.23782 (9)	0.0450 (4)	
C12	0.1848 (2)	0.52986 (17)	0.23845 (10)	0.0569 (5)	
H12	0.2175	0.5535	0.1910	0.068*	
C13	0.2253 (2)	0.62012 (16)	0.30772 (10)	0.0556 (5)	
H13	0.2847	0.7036	0.3061	0.067*	
C14	0.05407 (18)	0.31288 (16)	0.15997 (10)	0.0472 (4)	
C15	−0.03208 (19)	0.17588 (16)	0.15803 (10)	0.0519 (4)	
H15	−0.0451	0.1432	0.2058	0.062*	
C16	−0.09053 (17)	0.09935 (16)	0.09013 (10)	0.0478 (4)	
H16	−0.0694	0.1342	0.0437	0.057*	
C17	−0.18565 (17)	−0.03537 (16)	0.07925 (10)	0.0457 (4)	
C18	−0.23720 (19)	−0.09465 (18)	0.14335 (11)	0.0547 (5)	
H18	−0.2128	−0.0471	0.1950	0.066*	
C19	−0.3238 (2)	−0.22256 (18)	0.13200 (11)	0.0592 (5)	
H19	−0.3565	−0.2604	0.1758	0.071*	
C20	−0.36168 (19)	−0.29412 (17)	0.05589 (11)	0.0537 (4)	
H20	−0.4188	−0.3808	0.0475	0.064*	
C21	−0.31308 (17)	−0.23433 (16)	−0.00706 (10)	0.0468 (4)	
C22	−0.22641 (17)	−0.10648 (15)	0.00247 (10)	0.0452 (4)	
H22	−0.1960	−0.0688	−0.0418	0.054*	
C24	0.2995 (2)	1.36508 (16)	0.59909 (10)	0.0515 (4)	
C25	0.16002 (19)	1.30893 (16)	0.57121 (10)	0.0525 (4)	
H25	0.0933	1.3279	0.6012	0.063*	
C26	0.12012 (18)	1.22493 (15)	0.49902 (10)	0.0494 (4)	
H26	0.0264	1.1889	0.4804	0.059*	
C27	0.21752 (18)	1.19291 (15)	0.45338 (9)	0.0450 (4)	
C28	0.3555 (2)	1.25126 (17)	0.48255 (11)	0.0591 (5)	
H28	0.4227	1.2328	0.4528	0.071*	
C29	0.3969 (2)	1.33651 (17)	0.55475 (11)	0.0614 (5)	
H29	0.4907	1.3741	0.5730	0.074*	
C30	0.17366 (18)	1.09760 (15)	0.37753 (9)	0.0457 (4)	
C31	0.2555 (2)	1.10493 (17)	0.31492 (11)	0.0616 (5)	
H31	0.3384	1.1733	0.3197	0.074*	
C32	0.2167 (2)	1.01329 (17)	0.24594 (11)	0.0608 (5)	
H32	0.2732	1.0211	0.2048	0.073*	
C33	0.09445 (18)	0.90955 (15)	0.23713 (9)	0.0470 (4)	
C34	0.01134 (18)	0.90247 (16)	0.29847 (9)	0.0512 (4)	
H34	−0.0715	0.8340	0.2935	0.061*	
C35	0.04910 (19)	0.99518 (16)	0.36696 (10)	0.0516 (4)	
H35	−0.0097	0.9892	0.4069	0.062*	
C36	0.05532 (18)	0.81439 (17)	0.16040 (10)	0.0489 (4)	
C37	−0.03874 (19)	0.68039 (16)	0.16045 (10)	0.0524 (4)	
H37	−0.0619	0.6536	0.2089	0.063*	
C38	−0.09019 (17)	0.59839 (16)	0.09282 (10)	0.0480 (4)	
H38	−0.0623	0.6286	0.0460	0.058*	
C39	−0.18650 (17)	0.46484 (15)	0.08366 (10)	0.0452 (4)	
C40	−0.23020 (17)	0.39373 (15)	0.00693 (10)	0.0450 (4)	
H40	−0.2002	0.4310	−0.0375	0.054*	
C41	−0.31891 (17)	0.26675 (15)	−0.00239 (10)	0.0453 (4)	
C42	−0.36545 (18)	0.20710 (16)	0.06113 (11)	0.0523 (4)	
H42	−0.4239	0.1210	0.0531	0.063*	
C43	−0.3234 (2)	0.27813 (18)	0.13724 (11)	0.0568 (5)	
H43	−0.3546	0.2402	0.1812	0.068*	
C44	−0.23521 (19)	0.40527 (18)	0.14850 (11)	0.0534 (4)	
H44	−0.2078	0.4521	0.2002	0.064*	
O2	0.08909 (14)	0.35270 (12)	0.09847 (7)	0.0604 (4)	
O3	−0.33389 (15)	−0.25019 (13)	−0.14475 (8)	0.0693 (4)	
O4	−0.41772 (16)	−0.42643 (12)	−0.09601 (8)	0.0764 (4)	
O6	0.10085 (14)	0.84828 (12)	0.09975 (7)	0.0635 (4)	
O7	−0.34408 (15)	0.25180 (12)	−0.14019 (8)	0.0673 (4)	
O8	−0.42610 (15)	0.07543 (11)	−0.09182 (8)	0.0741 (4)	
N1	−0.35712 (15)	−0.30845 (14)	−0.08820 (9)	0.0552 (4)	
N2	−0.36572 (15)	0.19309 (14)	−0.08378 (9)	0.0537 (4)	
O1A	0.3604 (2)	0.9371 (2)	0.67405 (11)	0.0888 (6)	0.979 (4)
C1A	0.4232 (3)	1.0708 (3)	0.67750 (17)	0.1099 (12)	0.979 (4)
H1A	0.4480	1.1125	0.7324	0.165*	0.979 (4)
H1B	0.5050	1.0855	0.6504	0.165*	0.979 (4)
H1C	0.3598	1.1069	0.6520	0.165*	0.979 (4)
O1B	0.376 (8)	0.980 (4)	0.651 (4)	0.057 (9)	0.021 (4)
C1B	0.473 (8)	1.097 (5)	0.630 (5)	0.039 (14)	0.021 (4)
H1D	0.4976	1.1656	0.6756	0.058*	0.021 (4)
H1E	0.5549	1.0778	0.6150	0.058*	0.021 (4)
H1F	0.4308	1.1236	0.5862	0.058*	0.021 (4)
O5A	0.3198 (16)	1.4345 (17)	0.6756 (8)	0.065 (2)	0.55 (4)
C23A	0.4604 (13)	1.4924 (18)	0.7090 (8)	0.075 (3)	0.55 (4)
H23A	0.4644	1.5384	0.7620	0.113*	0.55 (4)
H23B	0.5031	1.4253	0.7113	0.113*	0.55 (4)
H23C	0.5089	1.5526	0.6762	0.113*	0.55 (4)
O5B	0.337 (2)	1.4596 (18)	0.6671 (10)	0.063 (3)	0.45 (4)
C23B	0.4823 (18)	1.5283 (19)	0.6918 (13)	0.096 (4)	0.45 (4)
H23D	0.4920	1.5904	0.7396	0.144*	0.45 (4)
H23E	0.5290	1.4667	0.7021	0.144*	0.45 (4)
H23F	0.5224	1.5735	0.6500	0.144*	0.45 (4)

### Atomic displacement parameters (Å^2^)

**Table:**

	*U*^11^	*U*^22^	*U*^33^	*U*^12^	*U*^13^	*U*^23^
C2	0.0602 (12)	0.0590 (11)	0.0466 (11)	0.0059 (9)	0.0057 (9)	−0.0083 (9)
C3	0.0922 (17)	0.0686 (13)	0.0458 (11)	0.0138 (11)	0.0041 (10)	0.0082 (9)
C4	0.0888 (15)	0.0490 (10)	0.0516 (11)	0.0129 (10)	0.0025 (10)	0.0069 (9)
C5	0.0424 (10)	0.0440 (8)	0.0437 (9)	0.0089 (7)	0.0033 (7)	0.0033 (7)
C6	0.0646 (12)	0.0471 (9)	0.0538 (11)	0.0131 (8)	0.0042 (9)	0.0061 (8)
C7	0.0739 (14)	0.0453 (10)	0.0647 (13)	0.0090 (9)	0.0078 (10)	−0.0028 (9)
C8	0.0453 (10)	0.0405 (8)	0.0426 (9)	0.0090 (7)	0.0017 (7)	0.0034 (7)
C9	0.0886 (15)	0.0483 (10)	0.0434 (10)	−0.0017 (10)	0.0161 (10)	0.0041 (8)
C10	0.0847 (15)	0.0411 (9)	0.0514 (11)	−0.0042 (9)	0.0132 (10)	0.0013 (8)
C11	0.0460 (10)	0.0441 (9)	0.0410 (9)	0.0112 (7)	0.0007 (7)	0.0028 (7)
C12	0.0649 (12)	0.0543 (10)	0.0405 (10)	0.0031 (9)	0.0061 (8)	0.0079 (8)
C13	0.0619 (12)	0.0430 (9)	0.0472 (10)	−0.0042 (8)	0.0048 (9)	0.0063 (8)
C14	0.0450 (10)	0.0507 (9)	0.0413 (9)	0.0127 (8)	0.0006 (8)	−0.0004 (7)
C15	0.0590 (11)	0.0481 (9)	0.0425 (10)	0.0122 (8)	−0.0001 (8)	0.0009 (8)
C16	0.0445 (10)	0.0491 (9)	0.0450 (10)	0.0115 (8)	0.0032 (8)	0.0009 (7)
C17	0.0401 (9)	0.0484 (9)	0.0455 (9)	0.0144 (7)	0.0005 (7)	−0.0012 (7)
C18	0.0552 (11)	0.0582 (11)	0.0462 (10)	0.0155 (9)	0.0027 (8)	0.0011 (8)
C19	0.0619 (12)	0.0617 (11)	0.0525 (11)	0.0160 (9)	0.0098 (9)	0.0108 (9)
C20	0.0476 (11)	0.0494 (9)	0.0619 (12)	0.0120 (8)	0.0067 (9)	0.0097 (9)
C21	0.0412 (9)	0.0460 (9)	0.0482 (10)	0.0131 (7)	−0.0018 (8)	−0.0034 (8)
C22	0.0414 (9)	0.0465 (9)	0.0439 (9)	0.0110 (7)	0.0035 (7)	0.0022 (7)
C24	0.0605 (12)	0.0462 (9)	0.0424 (10)	0.0145 (8)	0.0001 (8)	−0.0023 (7)
C25	0.0587 (12)	0.0510 (9)	0.0467 (10)	0.0149 (8)	0.0119 (8)	0.0075 (8)
C26	0.0503 (11)	0.0447 (9)	0.0479 (10)	0.0076 (8)	0.0074 (8)	0.0066 (7)
C27	0.0495 (10)	0.0382 (8)	0.0406 (9)	0.0047 (7)	0.0065 (8)	0.0041 (7)
C28	0.0541 (12)	0.0586 (11)	0.0554 (11)	0.0106 (9)	0.0095 (9)	−0.0066 (9)
C29	0.0522 (12)	0.0602 (11)	0.0597 (12)	0.0094 (9)	−0.0018 (9)	−0.0076 (9)
C30	0.0526 (10)	0.0388 (8)	0.0398 (9)	0.0081 (7)	0.0037 (8)	0.0018 (7)
C31	0.0618 (12)	0.0499 (10)	0.0529 (11)	−0.0076 (9)	0.0165 (9)	−0.0052 (8)
C32	0.0665 (13)	0.0577 (11)	0.0462 (10)	0.0032 (9)	0.0187 (9)	0.0003 (8)
C33	0.0527 (11)	0.0421 (8)	0.0390 (9)	0.0079 (7)	0.0005 (8)	0.0014 (7)
C34	0.0499 (11)	0.0478 (9)	0.0428 (10)	−0.0003 (8)	0.0026 (8)	0.0015 (8)
C35	0.0534 (11)	0.0523 (10)	0.0391 (9)	0.0030 (8)	0.0088 (8)	0.0037 (7)
C36	0.0487 (10)	0.0525 (10)	0.0405 (9)	0.0132 (8)	−0.0005 (8)	−0.0005 (8)
C37	0.0597 (12)	0.0472 (9)	0.0435 (10)	0.0115 (8)	0.0011 (8)	−0.0003 (8)
C38	0.0455 (10)	0.0501 (9)	0.0442 (9)	0.0132 (8)	0.0029 (8)	−0.0004 (7)
C39	0.0428 (9)	0.0451 (9)	0.0446 (9)	0.0133 (7)	0.0027 (7)	0.0000 (7)
C40	0.0431 (9)	0.0438 (8)	0.0448 (9)	0.0105 (7)	0.0050 (7)	0.0033 (7)
C41	0.0412 (9)	0.0439 (9)	0.0466 (10)	0.0107 (7)	0.0042 (7)	−0.0001 (7)
C42	0.0465 (10)	0.0464 (9)	0.0628 (12)	0.0114 (8)	0.0103 (8)	0.0100 (8)
C43	0.0586 (12)	0.0617 (11)	0.0504 (11)	0.0160 (9)	0.0139 (9)	0.0142 (9)
C44	0.0534 (11)	0.0595 (11)	0.0435 (10)	0.0166 (9)	0.0033 (8)	−0.0003 (8)
O2	0.0658 (9)	0.0619 (8)	0.0424 (7)	0.0077 (6)	0.0045 (6)	0.0012 (6)
O3	0.0807 (10)	0.0611 (8)	0.0508 (8)	0.0052 (7)	−0.0031 (7)	0.0031 (7)
O4	0.0864 (11)	0.0473 (7)	0.0706 (9)	−0.0072 (7)	0.0025 (8)	−0.0053 (6)
O6	0.0678 (9)	0.0671 (8)	0.0428 (7)	0.0063 (7)	0.0086 (6)	−0.0002 (6)
O7	0.0798 (10)	0.0566 (7)	0.0487 (8)	0.0003 (7)	−0.0002 (7)	0.0041 (6)
O8	0.0830 (10)	0.0426 (7)	0.0729 (9)	−0.0092 (7)	0.0131 (8)	−0.0048 (6)
N1	0.0491 (9)	0.0503 (8)	0.0559 (10)	0.0060 (7)	0.0001 (7)	−0.0001 (7)
N2	0.0496 (9)	0.0458 (8)	0.0554 (9)	0.0038 (7)	0.0061 (7)	−0.0002 (7)
O1A	0.1069 (14)	0.0754 (11)	0.0545 (10)	−0.0017 (10)	0.0064 (9)	−0.0168 (9)
C1A	0.119 (2)	0.0868 (18)	0.0797 (19)	−0.0156 (16)	0.0249 (17)	−0.0304 (15)
O1B	0.066 (11)	0.049 (11)	0.052 (12)	0.015 (8)	0.008 (9)	−0.003 (8)
C1B	0.053 (19)	0.020 (17)	0.034 (19)	0.003 (14)	0.002 (15)	−0.007 (14)
O5A	0.072 (4)	0.064 (5)	0.044 (3)	0.012 (3)	0.001 (2)	−0.013 (3)
C23A	0.062 (4)	0.093 (6)	0.052 (4)	0.013 (4)	−0.001 (3)	−0.018 (4)
O5B	0.068 (4)	0.059 (5)	0.049 (4)	0.009 (3)	0.002 (3)	−0.010 (3)
C23B	0.101 (7)	0.081 (6)	0.067 (6)	−0.012 (5)	−0.007 (5)	−0.021 (5)

### Geometric parameters (Å, º)

**Table:**

C2—C7	1.368 (3)	C28—C29	1.386 (2)
C2—C3	1.372 (3)	C28—H28	0.9300
C2—O1A	1.373 (2)	C29—H29	0.9300
C2—O1B	1.39 (2)	C30—C31	1.389 (2)
C3—C4	1.379 (2)	C30—C35	1.392 (2)
C3—H3	0.9300	C31—C32	1.378 (2)
C4—C5	1.384 (2)	C31—H31	0.9300
C4—H4	0.9300	C32—C33	1.386 (2)
C5—C6	1.380 (2)	C32—H32	0.9300
C5—C8	1.482 (2)	C33—C34	1.380 (2)
C6—C7	1.393 (2)	C33—C36	1.495 (2)
C6—H6	0.9300	C34—C35	1.377 (2)
C7—H7	0.9300	C34—H34	0.9300
C8—C9	1.384 (2)	C35—H35	0.9300
C8—C13	1.392 (2)	C36—O6	1.213 (2)
C9—C10	1.376 (2)	C36—C37	1.480 (2)
C9—H9	0.9300	C37—C38	1.320 (2)
C10—C11	1.380 (2)	C37—H37	0.9300
C10—H10	0.9300	C38—C39	1.465 (2)
C11—C12	1.383 (2)	C38—H38	0.9300
C11—C14	1.492 (2)	C39—C40	1.389 (2)
C12—C13	1.374 (2)	C39—C44	1.396 (2)
C12—H12	0.9300	C40—C41	1.382 (2)
C13—H13	0.9300	C40—H40	0.9300
C14—O2	1.2176 (19)	C41—C42	1.371 (2)
C14—C15	1.476 (2)	C41—N2	1.467 (2)
C15—C16	1.311 (2)	C42—C43	1.378 (2)
C15—H15	0.9300	C42—H42	0.9300
C16—C17	1.469 (2)	C43—C44	1.380 (2)
C16—H16	0.9300	C43—H43	0.9300
C17—C22	1.387 (2)	C44—H44	0.9300
C17—C18	1.391 (2)	O3—N1	1.2181 (18)
C18—C19	1.381 (2)	O4—N1	1.2266 (18)
C18—H18	0.9300	O7—N2	1.2176 (18)
C19—C20	1.378 (2)	O8—N2	1.2230 (17)
C19—H19	0.9300	O1A—C1A	1.390 (3)
C20—C21	1.369 (2)	C1A—H1A	0.9600
C20—H20	0.9300	C1A—H1B	0.9600
C21—C22	1.382 (2)	C1A—H1C	0.9600
C21—N1	1.463 (2)	O1B—C1B	1.45 (2)
C22—H22	0.9300	C1B—H1D	0.9600
C24—C29	1.367 (3)	C1B—H1E	0.9600
C24—O5A	1.377 (11)	C1B—H1F	0.9600
C24—O5B	1.382 (13)	O5A—C23A	1.421 (12)
C24—C25	1.386 (3)	C23A—H23A	0.9600
C25—C26	1.379 (2)	C23A—H23B	0.9600
C25—H25	0.9300	C23A—H23C	0.9600
C26—C27	1.394 (2)	O5B—C23B	1.442 (14)
C26—H26	0.9300	C23B—H23D	0.9600
C27—C28	1.380 (2)	C23B—H23E	0.9600
C27—C30	1.485 (2)	C23B—H23F	0.9600
			
C7—C2—C3	119.30 (17)	C24—C29—C28	119.73 (17)
C7—C2—O1A	125.5 (2)	C24—C29—H29	120.1
C3—C2—O1A	115.2 (2)	C28—C29—H29	120.1
C7—C2—O1B	99 (3)	C31—C30—C35	117.16 (14)
C3—C2—O1B	142 (3)	C31—C30—C27	121.80 (15)
C2—C3—C4	120.37 (18)	C35—C30—C27	121.03 (15)
C2—C3—H3	119.8	C32—C31—C30	121.49 (16)
C4—C3—H3	119.8	C32—C31—H31	119.3
C3—C4—C5	121.66 (17)	C30—C31—H31	119.3
C3—C4—H4	119.2	C31—C32—C33	120.80 (16)
C5—C4—H4	119.2	C31—C32—H32	119.6
C6—C5—C4	117.10 (15)	C33—C32—H32	119.6
C6—C5—C8	122.52 (15)	C34—C33—C32	118.16 (15)
C4—C5—C8	120.37 (15)	C34—C33—C36	123.01 (15)
C5—C6—C7	121.50 (17)	C32—C33—C36	118.76 (15)
C5—C6—H6	119.2	C35—C34—C33	121.06 (15)
C7—C6—H6	119.2	C35—C34—H34	119.5
C2—C7—C6	120.06 (17)	C33—C34—H34	119.5
C2—C7—H7	120.0	C34—C35—C30	121.29 (16)
C6—C7—H7	120.0	C34—C35—H35	119.4
C9—C8—C13	116.64 (14)	C30—C35—H35	119.4
C9—C8—C5	121.39 (15)	O6—C36—C37	121.55 (15)
C13—C8—C5	121.96 (14)	O6—C36—C33	119.61 (15)
C10—C9—C8	121.87 (16)	C37—C36—C33	118.84 (15)
C10—C9—H9	119.1	C38—C37—C36	120.99 (16)
C8—C9—H9	119.1	C38—C37—H37	119.5
C9—C10—C11	121.15 (15)	C36—C37—H37	119.5
C9—C10—H10	119.4	C37—C38—C39	126.95 (16)
C11—C10—H10	119.4	C37—C38—H38	116.5
C10—C11—C12	117.47 (15)	C39—C38—H38	116.5
C10—C11—C14	124.09 (15)	C40—C39—C44	118.48 (15)
C12—C11—C14	118.41 (15)	C40—C39—C38	118.47 (15)
C13—C12—C11	121.38 (16)	C44—C39—C38	123.05 (15)
C13—C12—H12	119.3	C41—C40—C39	119.00 (15)
C11—C12—H12	119.3	C41—C40—H40	120.5
C12—C13—C8	121.48 (15)	C39—C40—H40	120.5
C12—C13—H13	119.3	C42—C41—C40	122.76 (15)
C8—C13—H13	119.3	C42—C41—N2	118.71 (14)
O2—C14—C15	120.71 (15)	C40—C41—N2	118.52 (15)
O2—C14—C11	119.49 (15)	C41—C42—C43	118.25 (16)
C15—C14—C11	119.79 (15)	C41—C42—H42	120.9
C16—C15—C14	121.14 (16)	C43—C42—H42	120.9
C16—C15—H15	119.4	C42—C43—C44	120.35 (16)
C14—C15—H15	119.4	C42—C43—H43	119.8
C15—C16—C17	127.19 (16)	C44—C43—H43	119.8
C15—C16—H16	116.4	C43—C44—C39	121.14 (16)
C17—C16—H16	116.4	C43—C44—H44	119.4
C22—C17—C18	118.48 (15)	C39—C44—H44	119.4
C22—C17—C16	119.16 (15)	O3—N1—O4	123.03 (15)
C18—C17—C16	122.36 (15)	O3—N1—C21	118.70 (14)
C19—C18—C17	121.45 (16)	O4—N1—C21	118.26 (16)
C19—C18—H18	119.3	O7—N2—O8	122.92 (15)
C17—C18—H18	119.3	O7—N2—C41	118.70 (13)
C20—C19—C18	120.03 (17)	O8—N2—C41	118.38 (15)
C20—C19—H19	120.0	C2—O1A—C1A	117.9 (2)
C18—C19—H19	120.0	O1A—C1A—H1A	109.5
C21—C20—C19	118.24 (16)	O1A—C1A—H1B	109.5
C21—C20—H20	120.9	H1A—C1A—H1B	109.5
C19—C20—H20	120.9	O1A—C1A—H1C	109.5
C20—C21—C22	122.99 (15)	H1A—C1A—H1C	109.5
C20—C21—N1	118.41 (15)	H1B—C1A—H1C	109.5
C22—C21—N1	118.59 (15)	C2—O1B—C1B	126 (4)
C21—C22—C17	118.78 (15)	O1B—C1B—H1D	109.5
C21—C22—H22	120.6	O1B—C1B—H1E	109.5
C17—C22—H22	120.6	H1D—C1B—H1E	109.5
C29—C24—O5A	127.8 (7)	O1B—C1B—H1F	109.5
C29—C24—O5B	121.2 (9)	H1D—C1B—H1F	109.5
C29—C24—C25	119.69 (15)	H1E—C1B—H1F	109.5
O5A—C24—C25	112.2 (7)	C24—O5A—C23A	115.4 (11)
O5B—C24—C25	118.8 (9)	O5A—C23A—H23A	109.5
C26—C25—C24	120.02 (17)	O5A—C23A—H23B	109.5
C26—C25—H25	120.0	H23A—C23A—H23B	109.5
C24—C25—H25	120.0	O5A—C23A—H23C	109.5
C25—C26—C27	121.29 (17)	H23A—C23A—H23C	109.5
C25—C26—H26	119.4	H23B—C23A—H23C	109.5
C27—C26—H26	119.4	C24—O5B—C23B	119.2 (14)
C28—C27—C26	117.20 (15)	O5B—C23B—H23D	109.5
C28—C27—C30	121.75 (16)	O5B—C23B—H23E	109.5
C26—C27—C30	121.04 (15)	H23D—C23B—H23E	109.5
C27—C28—C29	122.06 (17)	O5B—C23B—H23F	109.5
C27—C28—H28	119.0	H23D—C23B—H23F	109.5
C29—C28—H28	119.0	H23E—C23B—H23F	109.5
			
C7—C2—C3—C4	1.1 (3)	C25—C24—C29—C28	−0.3 (3)
O1A—C2—C3—C4	−179.9 (2)	C27—C28—C29—C24	−0.2 (3)
O1B—C2—C3—C4	−172 (5)	C28—C27—C30—C31	−30.7 (3)
C2—C3—C4—C5	−0.9 (3)	C26—C27—C30—C31	150.72 (18)
C3—C4—C5—C6	0.3 (3)	C28—C27—C30—C35	148.07 (18)
C3—C4—C5—C8	179.23 (18)	C26—C27—C30—C35	−30.5 (3)
C4—C5—C6—C7	−0.1 (3)	C35—C30—C31—C32	−1.3 (3)
C8—C5—C6—C7	−178.97 (17)	C27—C30—C31—C32	177.48 (18)
C3—C2—C7—C6	−0.9 (3)	C30—C31—C32—C33	−0.6 (3)
O1A—C2—C7—C6	−179.72 (19)	C31—C32—C33—C34	1.5 (3)
O1B—C2—C7—C6	175 (3)	C31—C32—C33—C36	178.64 (18)
C5—C6—C7—C2	0.4 (3)	C32—C33—C34—C35	−0.6 (3)
C6—C5—C8—C9	−150.4 (2)	C36—C33—C34—C35	−177.52 (17)
C4—C5—C8—C9	30.8 (3)	C33—C34—C35—C30	−1.4 (3)
C6—C5—C8—C13	30.8 (3)	C31—C30—C35—C34	2.3 (3)
C4—C5—C8—C13	−148.06 (19)	C27—C30—C35—C34	−176.50 (16)
C13—C8—C9—C10	0.7 (3)	C34—C33—C36—O6	153.00 (19)
C5—C8—C9—C10	−178.14 (19)	C32—C33—C36—O6	−23.9 (3)
C8—C9—C10—C11	−0.1 (3)	C34—C33—C36—C37	−26.9 (3)
C9—C10—C11—C12	−0.5 (3)	C32—C33—C36—C37	156.11 (18)
C9—C10—C11—C14	−178.34 (19)	O6—C36—C37—C38	−8.6 (3)
C10—C11—C12—C13	0.5 (3)	C33—C36—C37—C38	171.31 (16)
C14—C11—C12—C13	178.48 (17)	C36—C37—C38—C39	−178.41 (17)
C11—C12—C13—C8	0.1 (3)	C37—C38—C39—C40	179.41 (17)
C9—C8—C13—C12	−0.7 (3)	C37—C38—C39—C44	−1.3 (3)
C5—C8—C13—C12	178.14 (18)	C44—C39—C40—C41	−0.6 (3)
C10—C11—C14—O2	172.29 (19)	C38—C39—C40—C41	178.74 (16)
C12—C11—C14—O2	−5.6 (3)	C39—C40—C41—C42	−0.4 (3)
C10—C11—C14—C15	−6.4 (3)	C39—C40—C41—N2	179.28 (14)
C12—C11—C14—C15	175.72 (17)	C40—C41—C42—C43	1.1 (3)
O2—C14—C15—C16	−10.5 (3)	N2—C41—C42—C43	−178.55 (15)
C11—C14—C15—C16	168.21 (16)	C41—C42—C43—C44	−0.9 (3)
C14—C15—C16—C17	−175.69 (17)	C42—C43—C44—C39	−0.1 (3)
C15—C16—C17—C22	−173.41 (17)	C40—C39—C44—C43	0.8 (3)
C15—C16—C17—C18	6.8 (3)	C38—C39—C44—C43	−178.47 (18)
C22—C17—C18—C19	1.9 (3)	C20—C21—N1—O3	167.83 (17)
C16—C17—C18—C19	−178.35 (18)	C22—C21—N1—O3	−11.0 (2)
C17—C18—C19—C20	−0.4 (3)	C20—C21—N1—O4	−11.1 (2)
C18—C19—C20—C21	−1.0 (3)	C22—C21—N1—O4	170.05 (16)
C19—C20—C21—C22	1.0 (3)	C42—C41—N2—O7	167.76 (17)
C19—C20—C21—N1	−177.82 (16)	C40—C41—N2—O7	−11.9 (2)
C20—C21—C22—C17	0.5 (3)	C42—C41—N2—O8	−11.5 (2)
N1—C21—C22—C17	179.29 (14)	C40—C41—N2—O8	168.82 (16)
C18—C17—C22—C21	−1.9 (3)	C7—C2—O1A—C1A	−14.0 (4)
C16—C17—C22—C21	178.34 (16)	C3—C2—O1A—C1A	167.1 (2)
C29—C24—C25—C26	−0.1 (3)	O1B—C2—O1A—C1A	−2 (8)
O5A—C24—C25—C26	−173.4 (9)	C7—C2—O1B—C1B	−35 (9)
O5B—C24—C25—C26	173.0 (11)	C3—C2—O1B—C1B	139 (6)
C24—C25—C26—C27	1.1 (3)	O1A—C2—O1B—C1B	155 (15)
C25—C26—C27—C28	−1.5 (2)	C29—C24—O5A—C23A	6.0 (16)
C25—C26—C27—C30	177.12 (15)	O5B—C24—O5A—C23A	−60 (6)
C26—C27—C28—C29	1.1 (3)	C25—C24—O5A—C23A	178.6 (9)
C30—C27—C28—C29	−177.54 (17)	C29—C24—O5B—C23B	−0.5 (19)
O5A—C24—C29—C28	171.8 (11)	O5A—C24—O5B—C23B	122 (7)
O5B—C24—C29—C28	−173.3 (11)	C25—C24—O5B—C23B	−173.6 (11)

### Hydrogen-bond geometry (Å, º)

**Table:**

*D*—H···*A*	*D*—H	H···*A*	*D*···*A*	*D*—H···*A*
C12—H12···O7^i^	0.93	2.42	3.185 (2)	139
C16—H16···O6^i^	0.93	2.48	3.349 (2)	156
C20—H20···O4^ii^	0.93	2.52	3.370 (2)	151
C23*A*—H23*A*···O4^iii^	0.96	2.55	3.356 (14)	142
C38—H38···O2^i^	0.93	2.49	3.361 (2)	156
C42—H42···O8^iv^	0.93	2.42	3.291 (2)	156

Symmetry codes: (i) −*x*, −*y*+1, −*z*; (ii) −*x*−1, −*y*−1, −*z*; (iii) *x*+1, *y*+2, *z*+1; (iv) −*x*−1, −*y*, −*z*.
